# Multi-faceted CRISPR/Cas technological innovation aspects in the framework of 3P medicine

**DOI:** 10.1007/s13167-023-00324-6

**Published:** 2023-05-22

**Authors:** Vincent Lučanský, Veronika Holubeková, Zuzana Kolková, Erika Halašová, Marek Samec, Olga Golubnitschaja

**Affiliations:** 1 Jessenius Faculty of Medicine in Martin (JFMED CU), Biomedical Center, Comenius University in Bratislava, Martin, Slovakia; 2 Department of Pathophysiology, Jessenius Faculty of Medicine, Comenius University in Bratislava, Martin, Slovakia; 3 Predictive, Preventive, Personalised (3P) Medicine, Department of Radiation Oncology, University Hospital Bonn, Rheinische Friedrich-Wilhelms-Universität Bonn, 53127 Bonn, Germany

**Keywords:** Predictive preventive personalized medicine (PPPM / 3PM), Primary & secondary care, CRISPR/Cas gene-editing technology, Defective genes, Restoration, Physiologic gene expression, Diagnostics, Therapy, COVID-19, Innovation, Health-to-disease transition, Human germline gene editing, Ethics, Mitochondrial disease, Disease severity, Health policy

## Abstract

Since 2009, the European Association for Predictive, Preventive and Personalised Medicine (EPMA, Brussels) promotes the paradigm change from reactive approach to predictive, preventive, and personalized medicine (PPPM/3PM) to protect individuals in sub-optimal health conditions from the health-to-disease transition, to increase life-quality of the affected patient cohorts improving, therefore, ethical standards and cost-efficacy of healthcare to great benefits of the society at large. The gene-editing technology utilizing CRISPR/Cas gene-editing approach has demonstrated its enormous value as a powerful tool in a broad spectrum of bio/medical research areas. Further, CRISPR/Cas gene-editing system is considered applicable to primary and secondary healthcare, in order to prevent disease spread and to treat clinically manifested disorders, involving diagnostics of SARS-Cov-2 infection and experimental treatment of COVID-19. Although the principle of the proposed gene editing is simple and elegant, there are a lot of technological challenges and ethical considerations to be solved prior to its broadly scaled clinical implementation. This article highlights technological innovation beyond the state of the art, exemplifies current achievements, discusses unsolved technological and ethical problems, and provides clinically relevant outlook in the framework of 3PM.

## Preamble

Reactive medicine as the currently implemented approach to treat clinically manifested disorders, has reached its limits from both ethical and economical points of view [1]. This can be illustrated by epidemics of non-communicable disorders demonstrating over a half of billion patients diagnosed with the diabetes mellitus and co-morbidities [2], breast cancer as the leading malignancy in female subpopulations [3], prostate cancer with the disease management costs annually increasing more rapidly than for any other cancer type [4] and alarming statistics of neurodegenerative disorders worldwide [5]**.** Since 2009, European Association for Predictive, Preventive and Personalised Medicine (EPMA, Brussels, www.epmanet.eu) promotes the paradigm change from reactive approach to predictive, preventive, and personalized medicine (PPPM/3PM) to reverse currently observed unfavourable trends, to protect individuals in sub-optimal health conditions from the health-to-disease transition, to increase life-quality of the affected patient cohorts improving, therefore, ethical standards and cost-efficacy of healthcare to great benefits of the society at large [6–8].

CRISPR/Cas gene-editing approach is applicable to primary and secondary healthcare, in order to prevent spread of diseases and to treat clinically manifested disorders. Although the principle of the proposed gene editing is simple and elegant, there are a lot of technological challenges and ethical considerations to be solved prior to its broadly scaled clinical implementation.

This article highlights technological innovation beyond the state of the art, exemplifies current achievements, discusses unsolved technological and ethical problems, and provides clinically relevant outlook in the framework of 3PM.

## Technological innovation by the CRISPR/Cas gene editing

CRISPR (Clustered Regularly Interspaced Short Palindromic Repeats)/Cas9 (CRISPR associated protein 9) system originated from bacteria and archaea, providing an adaptive immune response against viruses and plasmids. This defense system uses small RNA sequences of pathogen origin for sequence-specific detection and silencing of foreign, invading nucleic acids [9]. Although the Cas9 molecule is a part of prokaryotic defense mechanisms [10], it is fully functional in a broad spectrum of both prokaryotic and eukaryotic organisms.

A list of suitable target organisms and derived cell lines, that can be modified using the CRISPR/Cas9 system, covers the whole spectrum of life forms. Naturally, the most common are laboratory mammals such as mice [11–13], rats [14, 15], and rabbits [16], but also pigs [17, 18], dogs [19], and particularly human cell lines [20, 21]. Human gene editing is regulated by strict ethical norms and currently undergoes scientific, philosophical, and political discussion [22, 23]; however, in principle, it is executable. Besides the mammals, also genome of reptiles [24], amphibians (*Xenopus laevis* and *X. tropicalis*) [25, 26], fish (*Danio rerio*, *Oryzias latipes*) [26–28], insects (*Drosophila melanogaster*, mosquitoes) [29, 30], worms (*Caenorhabditis elegans*) [31, 32], plants [33–35], fungi [36, 37], bacteria [38], and viruses [39] can be edited by CRISPR/Cas9. Paradoxically, using the CRISPR/Cas9 technology is not as common in bacteria as in other organisms, likely because other methods based on homologous recombination were already available for efficient manipulation of their genomes [40].

The Cas molecules used for the gene editing originate from different organisms; thus, it is not surprising that these molecules differ in sequences as well as other properties and requirements (e.g., size of a gene, efficacy, target molecule type, or PAM sequence); however, more or less identical mechanism of action is shared by all [41]. Most frequently and first utilized is spCas9, originally isolated from *Staphylococcus pyogenes* [42–44]; later, other isolated Cas molecules from the other microorganisms started to be used as well. A list of examples contains *Staphylococcus aureus* [45, 46], *Francisella novicida* [47, 48], *Neisseria meningitides* [49], *Campylobacter jejuni* [50, 51], *Streptococcus thermophilus* [52, 53], *Acidaminococcus* spp. and *Lachnospiraceae bacterium* [54].

As mentioned above, the CRISPR/Cas system is commonly used in research studies to better understand biological processes. The development of precise treatment strategies should be preceded by the monitoring of genome editing experiments by NGS technology [55, 56] to eliminate unintended gene modifications[57]. This review focuses on CRISPR/Cas systems that are potentially utilized in personalized medicine.

### The brief history of CRISPR/Cas9 discovery

The discovery of the CRIPSR/Cas9 mechanism started in 1987, when an unusual repetitive DNA sequence with an unknown function, which was subsequently defined as a CRISPR, was described in the *Escherichia coli* genome [58]. Later, similar sequence patterns were reported in a range of other bacteria and archaea, suggesting an important role for such evolutionarily conserved clusters of repeated sequences. At the beginning of this century, several conserved genes regularly present adjacent to the CRISPR region were also discovered. These genes were named CRISPR-associated sequences (Cas). To date, six CRISPR–Cas types have been described, which feature highly diverse *cas* gene content and operon organization. However, the function of these sequences remained unclear at that time [39, 40]. In 2005, several groups described observation that CRISPR sequences share homology with phages and plasmids; moreover, these phages and plasmids do not infect host strains harboring the homologous spacer sequences in the CRISPR. These observations concluded that these sequences are involved in the framework of a biological defense system—an adaptive immunity evolved by bacteria to destroy viral pathogens by cutting specifically their DNA with Cas proteases [40, 59–61]. Finally, in 2012 first publication described genome-editing technology based on CRISPR/Cas9 [9]. Recently, in 2020, the first patient legally received gene-editing therapy with CRISPR/Cas9 directly administered into the body. This treatment aims to remove mutations that cause a rare genetic condition called Leber’s congenital amaurosis, causing blindness [62]. Such an approach can be used as a paradigm for the personalized treatment of other diseases with known genetic origin.

### General mechanism of CRISPR/Cas9 function

The CRISPR/Cas9 complex is an RNA-guided DNA endonuclease that recognizes target sites by RNA–DNA complementarity and produces sequence-specific double-stranded DNA break [63].

**Fig. 1:**
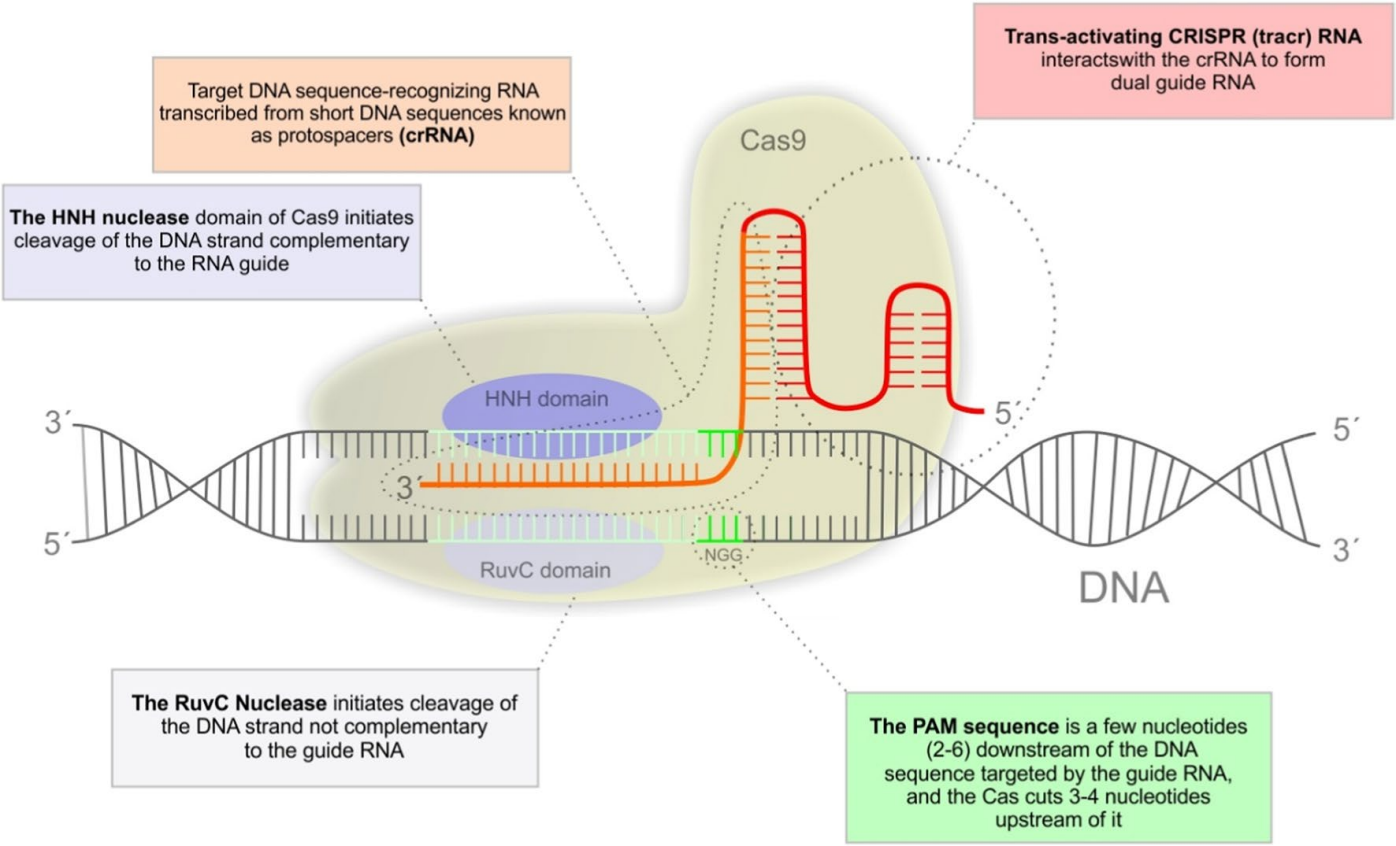
Schematic description of the general mechanism of CRISPR/Cas9 action

The CRISPR/Cas9 system consists of three key components: the CRISPR-associated DNA cleaving endonuclease Cas9 protein, a target DNA sequence-recognizing RNA transcribed from short DNA sequences known as protospacers (crRNA), and a trans-activating RNA (tracrRNA) [64]. The Cas9 nuclease contains two domains with endonuclease activity: RuvC and HNH, which together produces blunt double-stranded breaks. The DNA-binding crRNA recognized a 20-nucleotide-long DNA target sequence. The trans-activating crRNA (tracrRNA) anchors the crRNA to the Cas9 protein. Recognition and cleavage of a target site by Cas9 are conditioned and strictly depend on the presence of a so-called protospacer adjacent motif (PAM) sequence NGG immediately downstream of the crRNA target sequence. Cleavage occurs 3 bp upstream of the PAM [65]. The presence of a PAM sequence is the critical limitation of CRISPR/Cas9 gene editing. Currently, there are known various PAM sequences for different Cas molecules of different origins. Also, artificial Cas9 variants with expanded PAM compatibility were prepared [66]. Nowadays, the most common implementation of CRISPR genome editing in eukaryotic cells relies on a 2-component system consisting of Cas9 nuclease and a single chimeric guide RNA (sgRNA) that consist of crRNA and tracrRNA and provides both the recognition and structural function [65]. For the schematic visualization of the general mechanism of CRISPR/Cas9, see Fig. 1.

## CRISPR/Cas9 and other Cas molecule-based technologies are instrumental for 3P medicine

The CRISPR/cas9 system can improve human health by correcting genomes and searching for tumor manifestation, and capturing potential pathways of metastasis in organs. Similarly, in hereditary diseases, nucleases could be used to treat various diseases, including hematological and neurological disorders, autosomal dominant and X-linked disorders, and many others [67]. The basic principles of CRISPR/Cas technology, potentially useful in clinical practice, we discuss below. Generally, three main areas for CRISPR/Cas9 application in PPP are screening for inherited disease predispositions, diagnostic of molecular-based diseases, and therapy of such conditions.

### CRISPR/Cas9 gene knock-out of defective genes

**Fig. 2:**
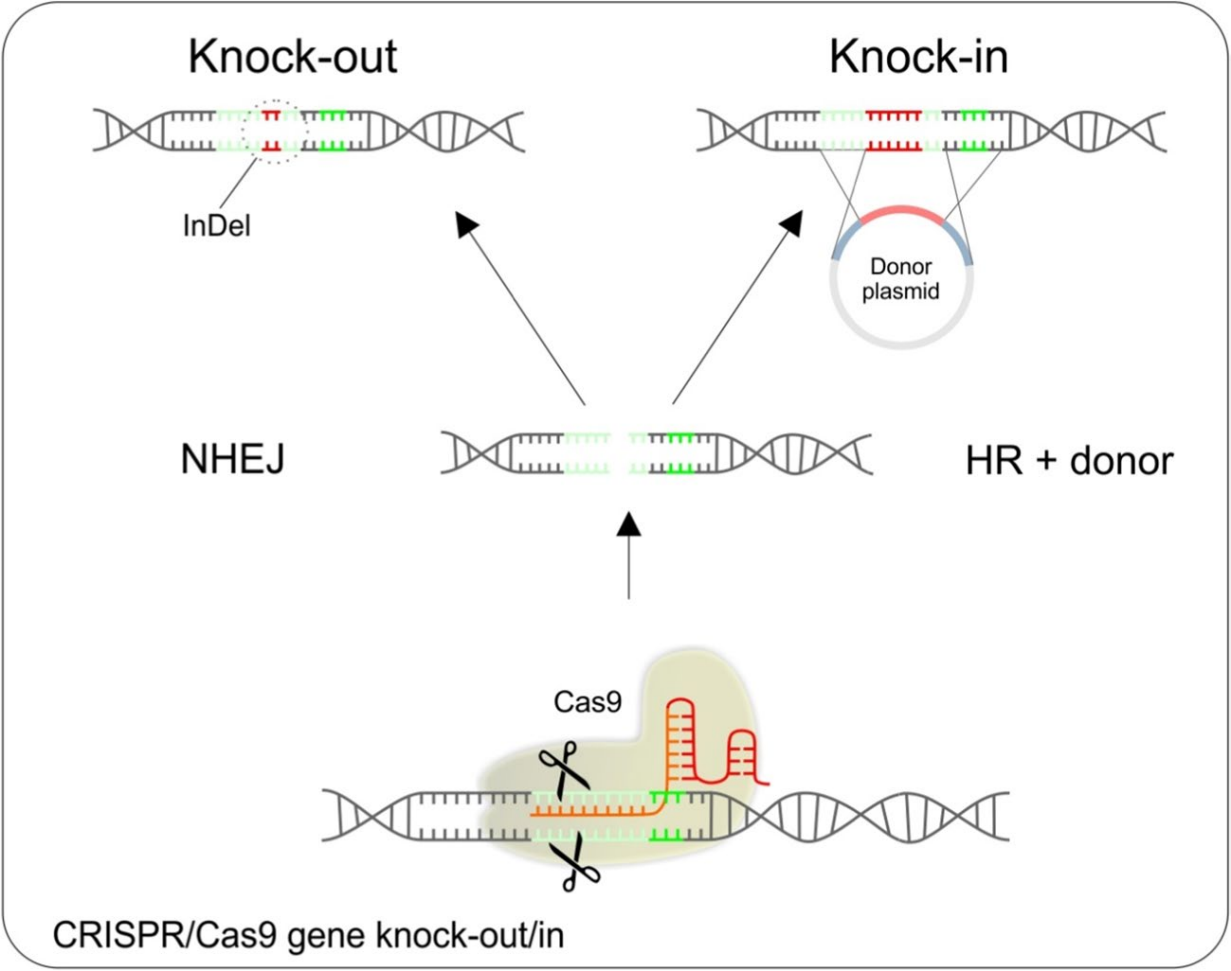
Schematic visualization of CRISPR/Cas9-mediated gene knock-out and knock-in

The CRISPR/Cas9 gene knock-out is a molecular genetics technique in which a target gene is inactivated. In principle, CRISPR/Cas9 gene knock-out is a combination of 2 different events. First, the CRISPR/Cas9 complex cleaves the target DNA sequence with a resulting blunt double-strand break that is subsequently repaired by the error-prone non-homologous end joining pathway (NHEJ), resulting in gene inactivation by the creation of frameshift alleles by random insertions or deletions (indels) [68, 69]. The creation of gene knockouts is one of the first and most widely used applications of the CRISPR–Cas9 system. Potentially therapeutic knockout of some genetic variants of genes involved in autoimmune disorders, such as multiple sclerosis, type 1 diabetes mellitus, psoriasis, rheumatoid arthritis, inflammatory bowel disease, systemic lupus erythematosus, and type 1 coeliac disease [70], shall be considered. For the schematic visualization of CRISPR/Cas9 knock-out, see Fig. 2.

### CRISPR/Cas9 gene knock-in for restoration of physiologic gene expression

Unlike the gene knock-out, CRISPR/Cas9 gene knock-in is an insertion of DNA sequence into the genome without disrupting the ORF. It starts (just like knock-out) with CRISPR/Cas9 targeted cleavage of target DNA sequence with subsequent targeted integration of a sequence via the homology-directed repair pathway, which uses a provided DNA template to repair the cleavage [71]. A donor DNA containing a sequence surrounding the cleavage site with an insert in the frame has to be provided. For the schematic visualization of CRISPR/Cas9 knock-in, see Fig. 2.

The scale of modifications used in basic research includes the introduction of a single nucleotide–point mutation, small tags (Flag-tag, His-tag), up to larger protein sequences, very often fluorescent proteins [72]. However, this approach was used in experiments in cell lines in vitro, as well as in humanized centrosomal protein 290 (CEP290) mice in vivo carrying the mutation in the CEP290 gene, responsible for Leber congenital amaurosis type 10. The results showed the normal expression of the *CEP290* gene and demonstrated the ability of CRISPR/Cas9 to edit somatic primate cells in vivo at levels that met the target therapeutic threshold. The authors suggest the application of the method for other inherited retinal disorders [73].

### Nickases—a safer approach for gene editing

**Fig. 3:**
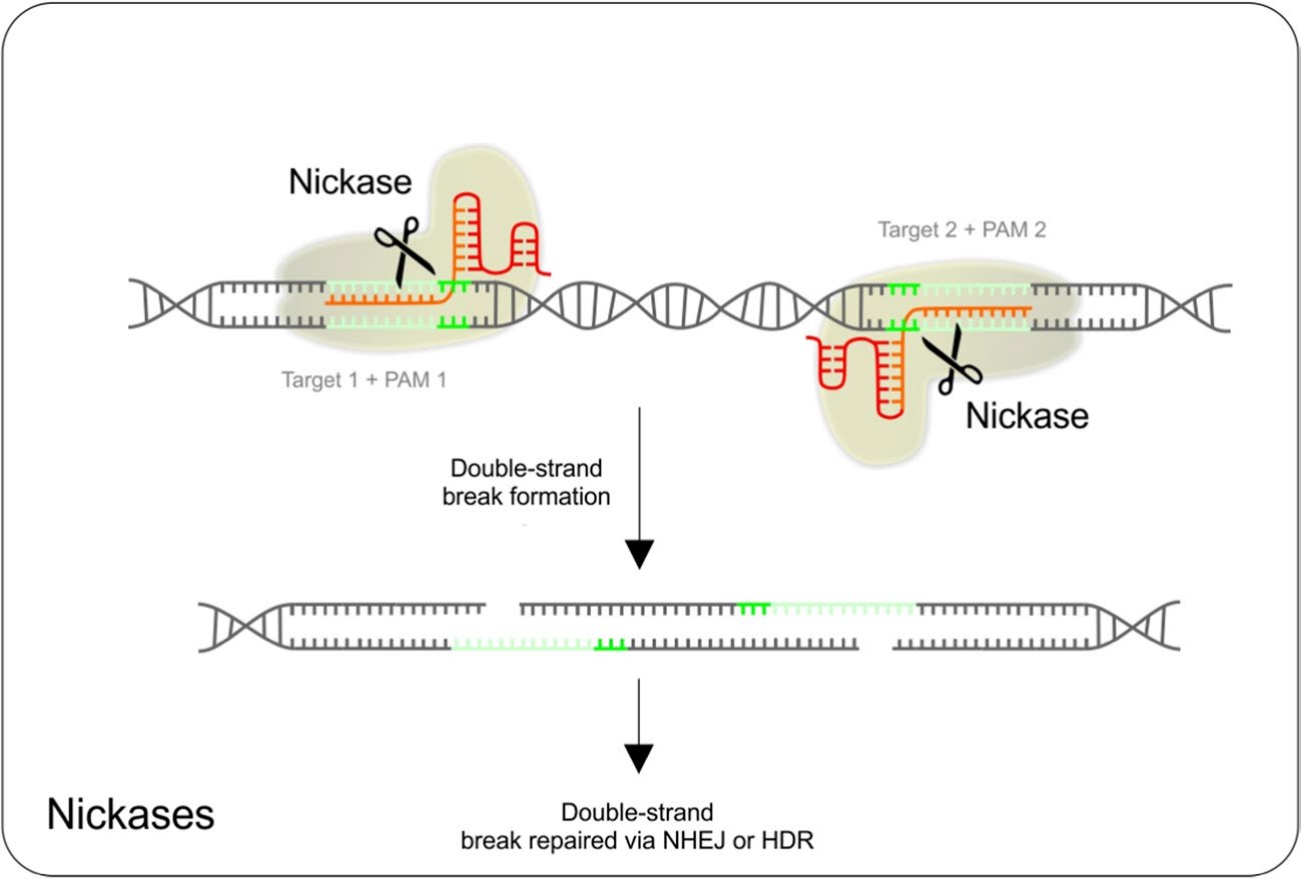
Schematic visualization of nickases mechanism of function

Off-targeting by CRISPR/Cas9 endonucleases is a significant concern. To address this problem, a paired nickase technology has been developed. To improve the specificity of CRISPR/Cas9 gene editing and to decrease the number of off-target mutagenesis events, CRISPR/Cas9 system was modified in the manner in which two single-strand nicks in proximity are created instead of a double-strand break. For this, a modified Cas9 protein called Cas9 nickase is used. Nickase is a catalytic mutant of Cas9 protein with one inactivated endonuclease domain; thus, it cleaves only one strand of the target dsDNA. When two different gRNAs targeting two neighboring sequences, however, on the opposite strands are used, then two separate single-strand breaks occur. This results in the formation of 5′ and 3′ overhangs at the cleaved target site. Induced break with overhang is either repaired by non-homologous end joining or homologous recombination-based repair mechanisms [74]. Using two close target sites on opposite strands decreases the possibility of random cleavage caused by off-target activity [75]. For the schematic visualization of nickases mechanism of function, see Fig. 3.

The catalytically dead dCas9 fused with the FokI domain works in an identical manner. The formation of DSB occurs only upon dimerization of FokI; thus, two unique gRNAs, binding to opposite strands approximately 15–25 bp apart, are necessary to bring two dCas9-FokI fusion proteins together to generate an active DNA-cleaving complex. This technology reduces the unwanted off-target activity of CRISPR, likewise the nickase approach [76, 77].

Also, nickases have been practically used in the development of sensitive and specific isothermal amplification of target genomic DNA adaptable in routine assays [78] and can potentially replace a traditional polymerase chain reaction (PCR) in non-laboratory on-site diagnostic applications [79].

### CRISPR/Cas9-mediated up- and downregulation of gene expression and epigenetic modifications

A direct downregulation of gene expression can be mediated by catalytically deactivated Cas9 (dCas9) with disturbed endonuclease activity. When co-expressed with a guide RNA, it generates a DNA recognition complex, targeting specific sequences, which can interfere with transcriptional elongation, RNA polymerase binding, or transcription factor binding. This system, called CRISPR interference (CRISPRi), can be used to repress multiple target genes simultaneously, and its effects are reversible [80].

The site-specific binding of dCas9 can be utilized also for specific emplacement of various activator or repressor domains fused with dCas9, which alters the gene expression, mostly via modification of methylation [81] and/or acetylation [82]. These effector domains can write or erase histone modifications or modulate DNA methylation. Epigenome editing can be used to transiently or stably activate or repress specific genes. It was demonstrated that a catalytic domain of the human demethylase TET1cd targeted to specific methylated regions can cause highly efficient demethylation, thus up-regulation of a particular gene and a heritable change of the phenotype. In combination with dCas9, it provides tools for the targeted removal of 5mC at specific loci in the genome with high specificity and minimal off-target effects [83].

On the other hand, the use of the dCas9 fused with DNA methyltransferase catalytic domain led to a reduced expression of the gene of interest and to change in the phenotype [84]. Indeed, DNA methylation plays a critical role in regulating gene expression. Dysregulation of DNA methylation is involved in the pathogenesis of numerous diseases. Thus, the potential of technologies designed to manipulate DNA methylation at specific genomic loci is very high [85].

In parallel, if dCas9 is linked to the histone-modifying domain, it edits the chromatin histone, resulting in either activation or deactivation of the gene of choice. For example, p300 histone acetyltransferase increases H3K27 acetylation, which leads to gene activation; SID4X decreases H3K27 acetylation, thus decreasing gene expression, KRAB domain increases methylation at H3K9me3 and subsequently silences the gene expression, and paradoxically, LSD1 decreases methylation at H3K4me and also repress the gene expression [82, 86].

**Fig. 4:**
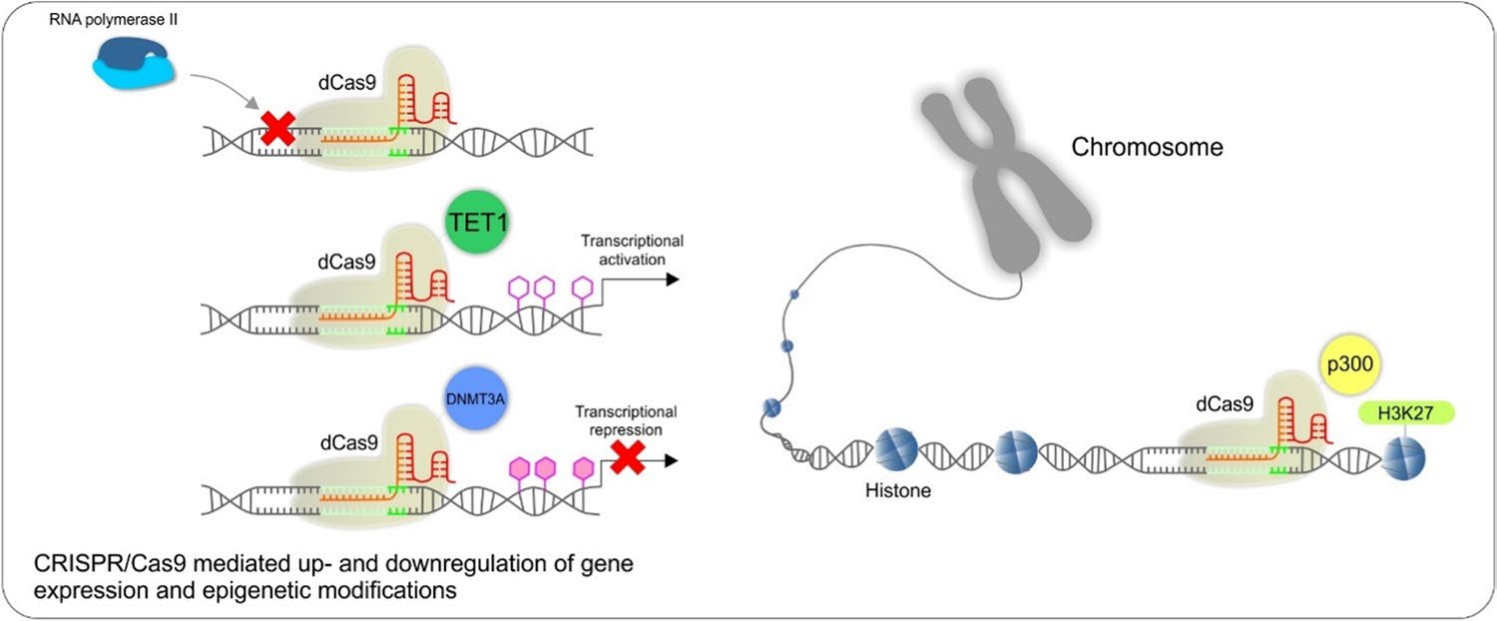
Examples of CRISPR/Cas9-mediated up- and downregulation of gene expression and epigenetic modifications

Epigenome editing tools have a place in precise medicine because they can be utilized in the treatment strategies of diseases, such as cancer, when some technological challenges of the method will be overcome [87]. Precise identification of epigenetically edited areas by NGS and elucidation of the interaction of edited genomes in epigenetic recombination should be taken into account [88]. For the schematic visualization of CRISPR/Cas9 utilization for up- and downregulation of gene expression and epigenetic modifications, see Fig. 4.

### Targeted base editing can be useful in personalized medicine of monogenic disease

A targeted base editing could represent a promising contribution to personalized medicine, especially since many diseases are caused by small nucleotide substitutions or deletions. The CRISPR/Cas9 base-editing system directly edits single nucleotides, which avoids the dependence on homology-dependent repair [89]. Either dCas9 or Cas9 nickase (nCas9) can be fused to deaminases to induce substitutions or base edits without inducing double-strand breaks; thus, this approach minimizes the indels. Deaminases combined with Cas9 nickase and uracil DNA glycosylase inhibitors can make precise edits, changing cytidine to thymidine with high efficiency [90].

**Fig. 5:**
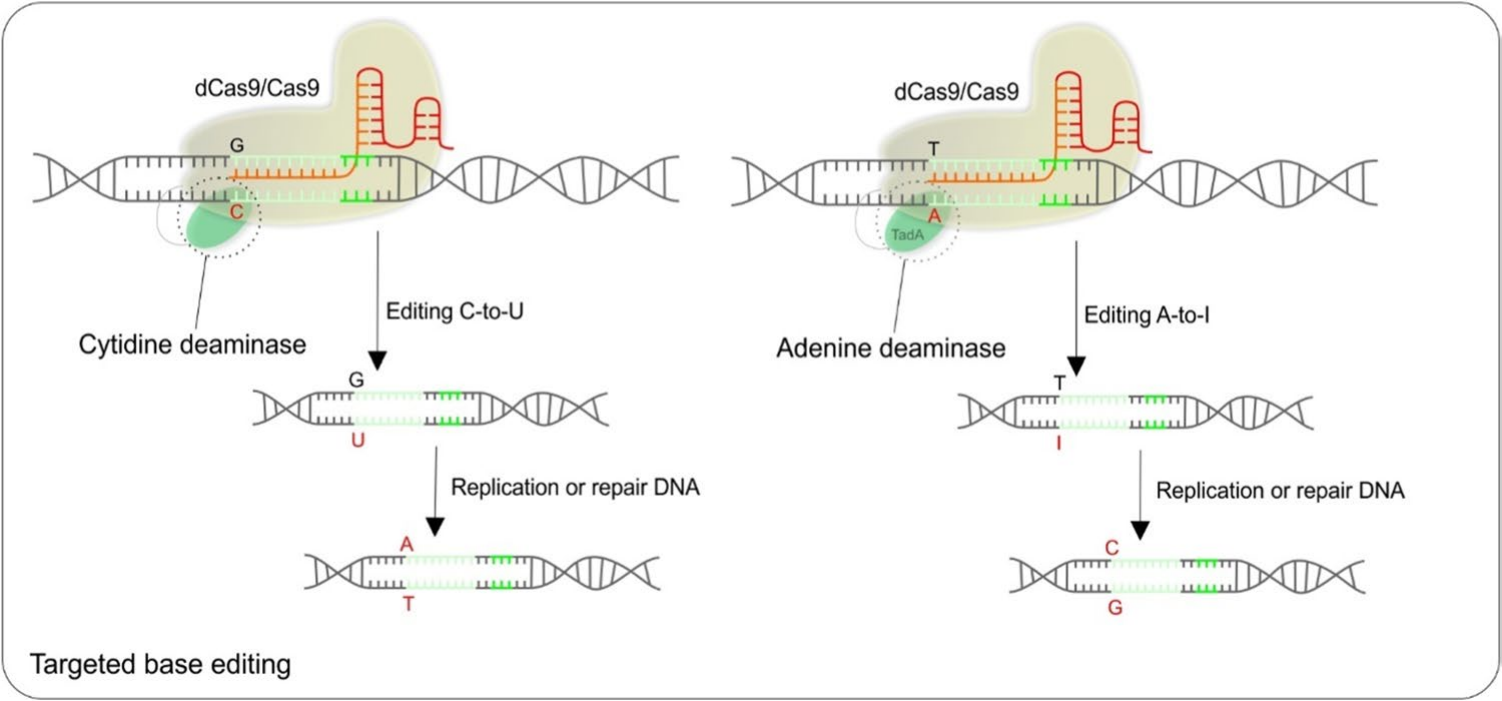
Examples of CRISPR/Cas9 targeted base editing

Until now, two kinds of base editors have been developed. The cytidine editor mediates the conversion of C/T (or G/A), whereas the adenosine editor affects an A/G (or T/C) substitution. The base editors are typically composed of a defective Cas9 protein (Cas9D10A or Cas9D10AH840A) and a deaminase fused to the Cas9 protein. Guided by the Cas9/sgRNA complex, the deaminase can be directed to any genomic locus to perform base editing in the single-stranded DNA (ssDNA) generated upon Cas9/sgRNA binding. By catalyzing the conversion of CAA, CAG, CGA, or TGG to TAA, TAG, or TGA codons, the cytidine base editor is capable of inactivating a target gene by generating a premature stop codon [91]. The base editors are applicable in the treatment of monogenic diseases with known pathogenic mutations and are intensively studied in experiments in vitro and in vivo. Actually, in vivo experiments showed that a single administration of base editors can have a longtime effect on many organs or tissues and improve the patient’s life that is complicated by symptomatic treatment. The rapid development of base editing tools is directly fused with the improvement in bioinformatic approaches that predict base editing outcomes. Once these shortcomings are eliminated, they can be used in personalized medicine [92]. For the schematic visualization of CRISPR/Cas9 targeted base editing, see Fig. 5.

### Imaging tools based on CRISPR/Cas9

**Fig. 6:**
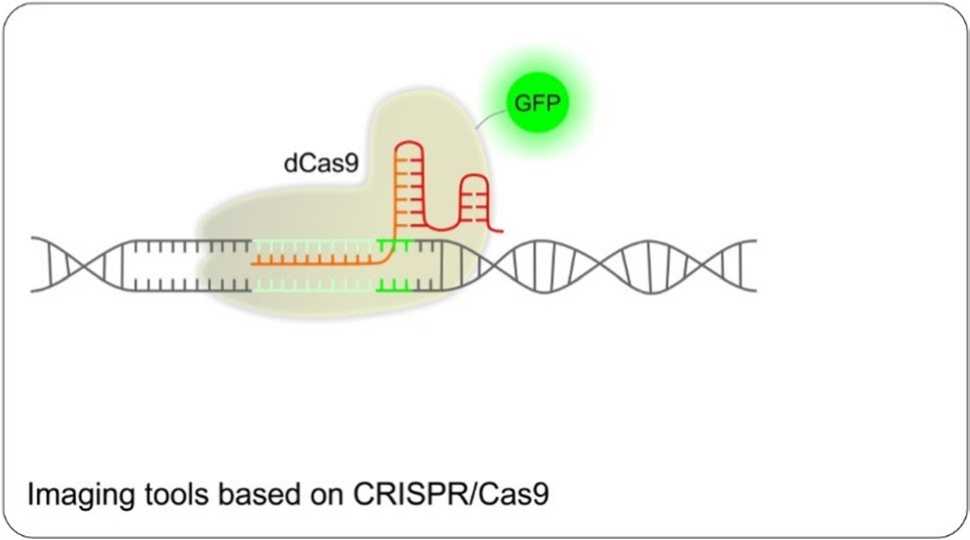
Utilization of dCas9 fused with a fluorescent protein to tag specific DNA sequence

Besides the utilization of standard CRISPR/Cas9 knock-in strategy to fuse DNA sequence coding for fluorescent proteins with the gene of interest, enabling the imaging of produced protein [93, 94], CRISPR/Cas9 technology can be utilized for direct labeling of DNA and/or RNA. The dCas9/gRNA complex tagged with one or several fluorescent molecules can label specific DNA loci in living cells [95] (see Fig. 6). This approach has been proven to be effective in monitoring the localization of any genomic sequence. In this direction, another advantageous method for enriching the signal in live cell imaging is the use of the dCas9-SunTag system [96]. In this case, dCas9 is fused to an epitope tail—a protein platform loaded with multiple epitopes, each of which is recognized by a single chain variable fragment (scFv) of antibody. Simultaneously, fluorescent molecules are fused to a single chain variable fragment (scFv). While the possible number of scFv epitopes is much higher than any possible number of fluorescent proteins that can be directly fused to dCas9, this approach outperforms previous ones when the intensity of the signal is taken into consideration [97]. As an example of an imaging method that is not based on the utilization of various fluorescent proteins, a Cas9-mediated fluorescence in situ hybridization (CASFISH) should be mentioned. In this case, Halo Tag conjugated to fluorescent dye was used as a shorter replacement for fluorescent proteins [98]. Another recently developed strategy is labeling viral DNA with streptavidin-conjugated Quantum Dots (QD) connected to dCas9-Bio/gRNA complex via streptavidin/biotin binding [99]. Imaging of target sequences based on CRISPR/Cas9 technology can be used in live cells to study native chromatin organization. The method is rapid, cost-effective, comfortable, and applicable to multiple targets, which gives the prerequisite for the inclusion of the method into the diagnostics of human diseases [98].

### RNA targeting by CRISPR/Cas systems

**Fig. 7:**
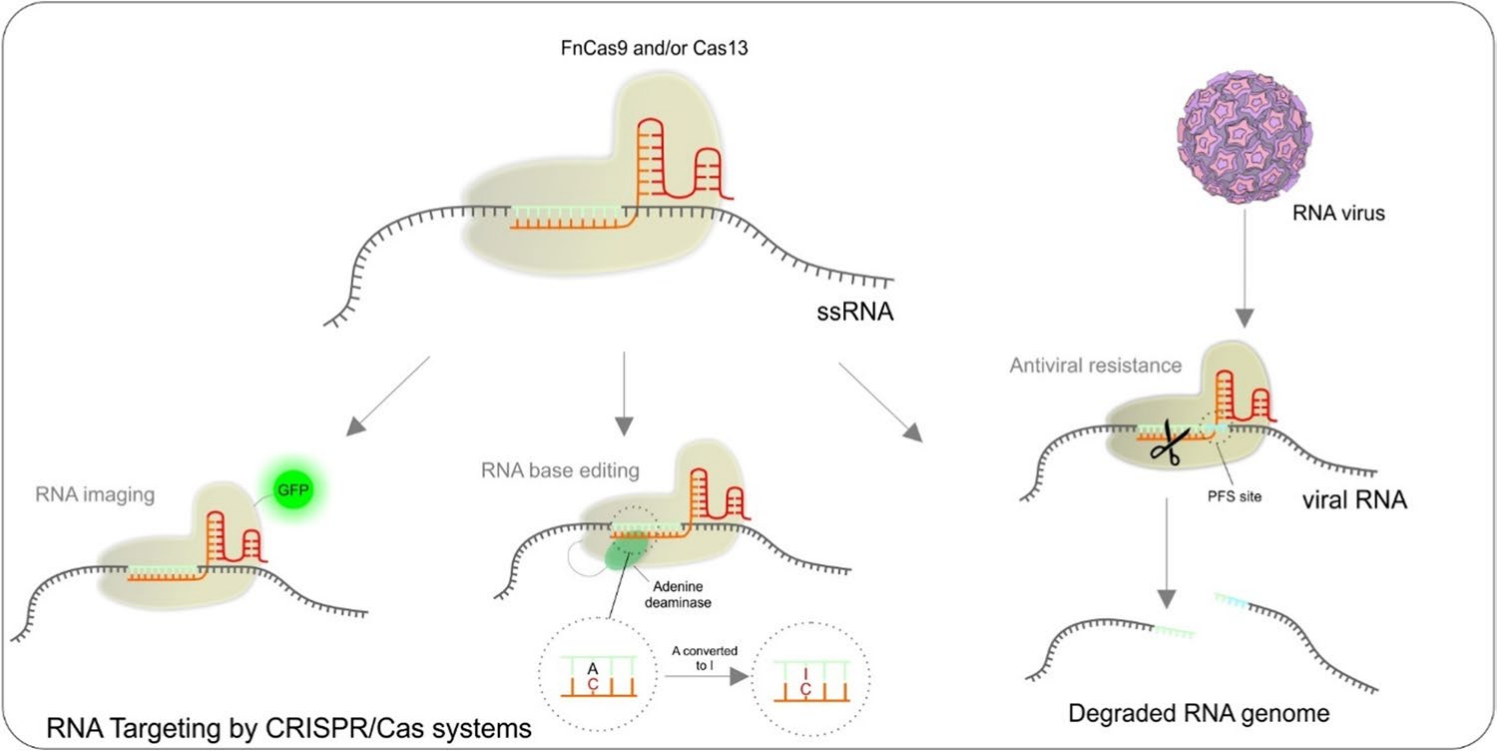
Possibilities of CRISPR/Cas techniques utilization for RNA targeting

Although CRISPR/Cas9 system in natural conditions recognizes and cleaves exclusively DNA molecules, it is also possible to target RNA molecules. Repurposing dCas9 of *S. pyogenes* to target RNA requires, besides the usual components: dCas9 and gRNA, also PAMmers—short PAM-presenting DNA oligonucleotides. Once used, it allows the Cas9 to bind with high affinity to the single-stranded RNA and subsequently perform its enzymatic activity [63]. Other options to target RNA are the usage of Cas9 molecules of *Francisella novicida* (FnCas9) and/or Cas13, which are known to recognize the RNA naturally [48, 100–102]. In parallel with Cas9-mediated technologies focused on DNA, there are similar alternatives for RNA, for example, imaging [103, 104], base editing of full-length transcripts to repair pathogenic mutations on mRNA level [101], potential experimental therapeutic applications against RNA viruses [48], the introduction of antiviral resistance in plants [105] or oncology research—via targeting of lncRNAs [106]. A recent pandemic of the SARS-CoV-2 RNA virus allowed the development of CRISPR/Cas12 and/Cas13 molecular diagnostic kits focused on viral RNA, running in isothermal conditions and with no requirement of special equipment or training people. Colorimetric and visual detection of the pathogen in samples allows massive use of this test type anywhere and is also adjustable for other viral agents [107] (see Fig. 7).

### Usage of CRISPR/Cas9 for the disease predisposition screening

Screening the disease as a part of preventive medicine represents a strategy to search for the risk markers or conditions that could turn into a disease in the future. Screening testing should be applied to individuals by cost-effective point-of-care diagnostics that advance the CRISPR/Cas system to candidate methods. The high-throughput genetic perturbation technologies are a useful tool for uncovering the involvement of particular genes in a broad spectrum of biological processes. Standard CRISPR/Cas9 knock-out combined with utilization of whole-genome or more specific gRNA libraries and subsequent identification of genes of interest represents a very effective approach for such screening [108]. Development of the CRISPR/Cas9-based loss-of-function screening assays enabled efficient identification of essential genes in mammalian cells involved in cancer therapy resistance [109], genes involved in the immune response to cancer [110], genes enabling the viral infection [111], genes associated with sepsis [112], biosynthesis pathways [113] and very probably many more.

In addition to screening based on a knock-out approach, catalytically inactive dCas9 fused with various transcriptional activator, repressor, and recruitment domains have been used to modulate gene expression without introducing irreversible mutations to the genome. In this case, gRNA libraries are focused on the upstream, regulating regions of investigated genes [114].

### Purification of specific target DNA with CRISPR/Cas9-based technology

The capability of dCas9 to bind selectively a specific DNA sequence is also employed in engineered DNA-binding molecule-mediated chromatin immunoprecipitation enChIP technology. The dCas9 molecule fused to an epitope tag is being used to isolate a given genomic locus. In addition, this technology allows for retaining the intracellular molecular interactions; thus, in vitro enChIP technology is of potential use not only for sequence-specific isolation of DNA but as well as for the identification of molecules interacting with genomic regions of interest [115, 116].

### The delivery strategy of the CRISPR/Cas9 system into target cells affects the outcome

Efficient and successful delivery of the CRISPR/Cas system into the target cells in vivo remains a challenge. Current delivery methods include viral and non-viral vectors and physical delivery. An effective delivery system must overcome the barriers of tissues and cell membranes; then, it should be transported to the nucleus, where it operates. The ineffective carriers may cause problems such as immune response, gene mutations, and triggering carcinogenesis. Another problem is associated with the size of packing molecules. However, nano-delivery systems seem to be efficient in experiments in vivo and in vitro [117].

**Fig. 8:**
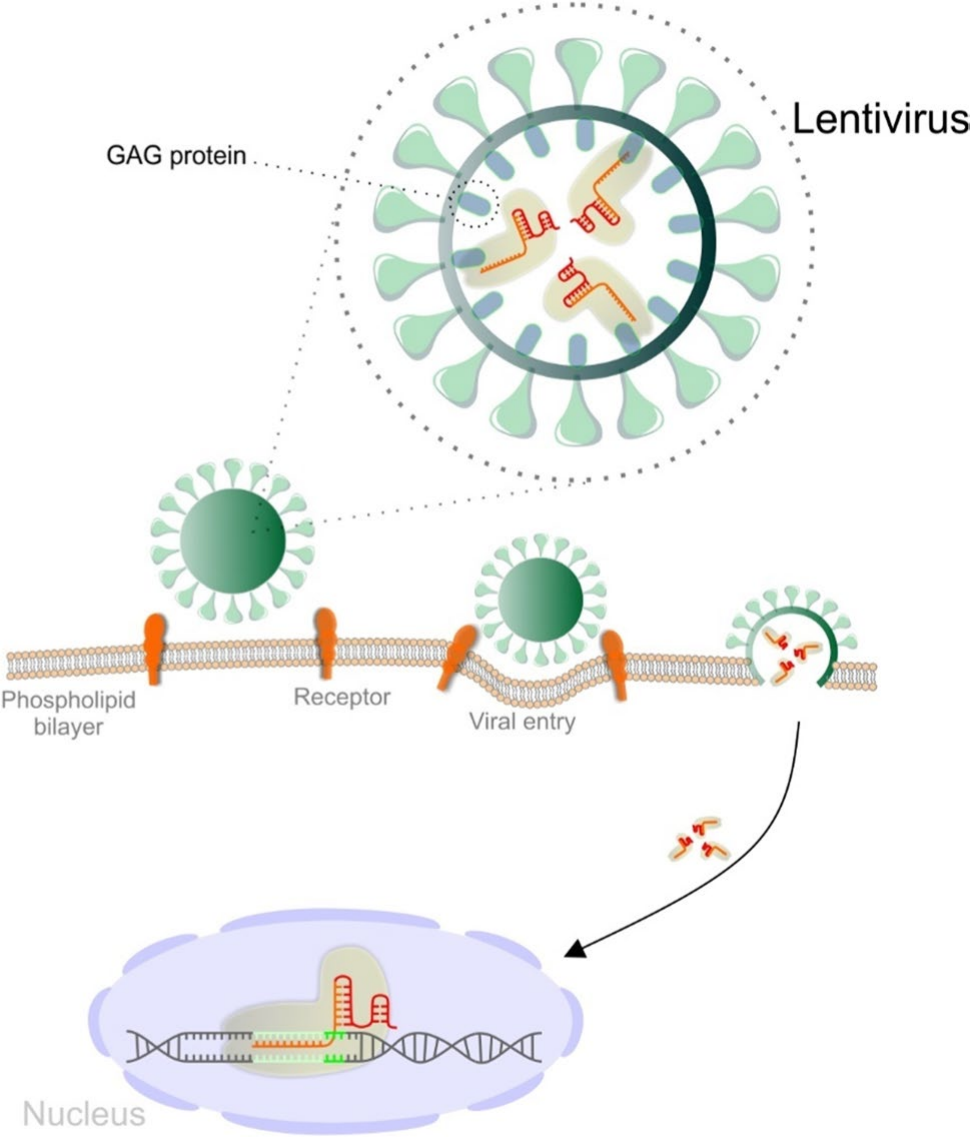
Delivery strategy for CRISPR/Cas9 system (nanoblade)

As we mentioned, the selection of a suitable delivery method for Cas9/sgRNA expressing cassettes into target cells is an important step. For mammalian cells, plasmid transfection is the most common way to deliver the Cas9/sgRNA-expressing cassette. However, transfection can cause overexpression of both Cas9 protein and sgRNA, and then induce off-target mutagenesis. In addition, many cell types are hard-to-transfect cells (such as hematopoietic cells, immune cells, stem cells, etc.). A microinjection is an option but with a low efficiency of only one cell per injection. Several groups used adenovirus to deliver Cas9/sgRNA expressing cassettes, but the construction and amplification of recombinant adenovirus were laborious and time-consuming. A single lentiviral vector was designed to simultaneously deliver Cas9 and sgRNA-expressing cassettes into the target cells. This system will enable genome editing in most cell types of interest [68]. Very promising is the so-called "nanoblade" technology [118], which avoids off-target mutation induced by the application of a standard CRIPSR/Cas9 system. This has been especially a significant concern when using this technique to generate a stable knock-out cell line [68]. To overcome this problem, engineered Murine leukemia virus-like particles loaded with Cas9-sgRNA ribonucleoproteins (Nanoblades) can be used for transduction and subsequent editing without integrating a virus genome, thus reducing editing to the one-time event [118]. The goal in the nanoparticle delivery will be a tissue-specific surface of nanostructure covered with specific ligands that will be suitable for clinical disease treatment [117]. Figure 8 represents nanoblade technology for CRISPR/Cas9 delivery.

## CRISPR/Cas-based diagnostics

The ability of gRNA-guided Cas molecules to recognize a specific sequence has its logical application in the diagnostics of various diseases. Indeed, several approaches to detect both specific DNA and RNA in samples have been developed recently. For this purpose, Cas12a, Cas13a, and Cas13b are employed most often. The action of these members of the Cas family differs from that of the Cas9. Unlike Cas9, upon the activation of these CRISPR/Cas complexes with a specific target, an indiscriminate cleavage of substrate nucleic acids occurs. In the diagnostic assays, substrate sequences are coupled to a fluorescent reporter. The fluorescence signal is activated only when the substrate is cleaved. The presence of a limited amount of target sequence can elicit intense fluorescence; thus, the methods based on these principles are very sensitive [119]. As an example, the DETECTR (DNA endonuclease-targeted CRISPR trans reporter) method can be mentioned. Isothermal Recombinase Polymerase Amplification (RPA) is coupled with subsequent Cas12a target DNA recognition, unleashing robust, indiscriminate cleavage of a quenched-fluorescent single-stranded DNA substrate reporter. This rapid, one-pot detection provides a straightforward platform for molecular diagnostics [120]. Another technique, named SHERLOCK (Specific High-sensitivity Enzymatic Reporter un-Locking), utilizes a combination of the isothermal RPA or reverse transcription (RT)-RPA with nuclease activity of Cas13a, followed by unspecific collateral cleavage of labeled reporter RNA resulting in signal release [121, 122]. In All-In-One Dual CRISPR/Cas12a (AIOD-CRISPR) assay, a pair of gRNAs was introduced to initiate dual CRISPR/Cas12a detection, which resulted in improved detection sensitivity. The nucleic acids (DNA and RNA) were successfully detected with a sensitivity of few copies, which is comparable with the real-time RT-PCR method [123]. Also, Cas9-based assays can be utilized for diagnostic purposes. In this case, for the identification of different strains. The combination of the isothermal amplification technique NASBA (Nucleic acid sequence-based amplification) and subsequent application of specific gRNA/Cas9 complex is used to differentiate strains in single-base discrimination. The result is either truncated or full-length DNA fragments, and only non-truncated fragments can trigger the toehold switch, leading to a color reaction [124].

After the emergence of the COVID-19 pandemic, various SARS-CoV-2 diagnostic techniques based on Cas12a and Cas13a have been developed by numerous research groups very rapidly, demonstrating the flexibility, adaptability, and versatility of this approach and its potential for future challenges [125–129].

**Fig. 9:**
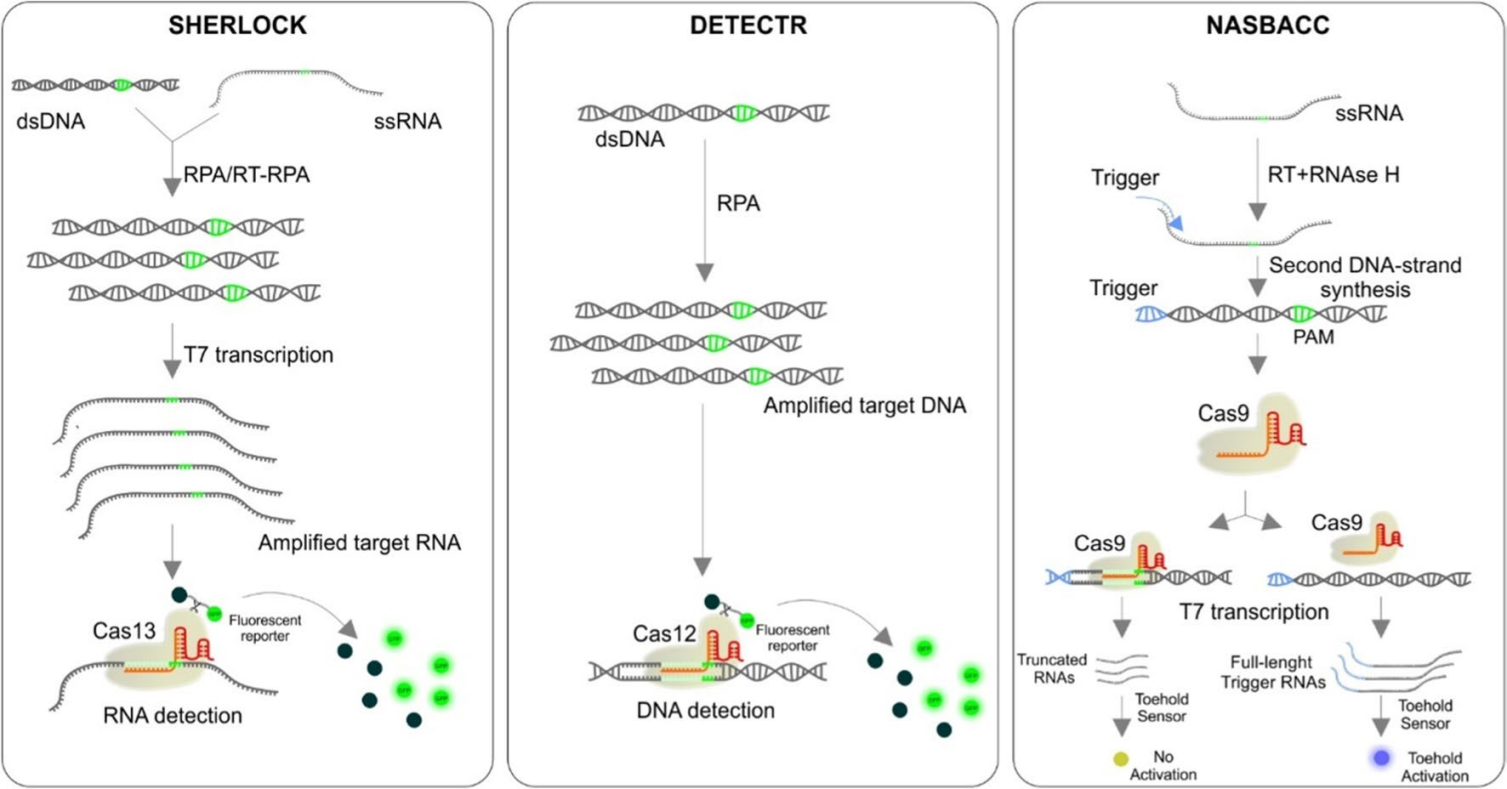
Examples of Cas molecules utilization for detection of different templates (RNA, DNA)

In general, the methods mentioned above and many similar methods have a great potential for developing next-generation point-of-care molecular diagnostics necessary for the development of personalized medicine. For examples of CRISPR/Cas-based detection methods, see Fig. 9.

## Fundamentals of the future CRISPR/Cas9-based therapy

The ability of CRISPR/Cas9-based methodology to modulate the expression of selected genes or precisely re-write the specific DNA sequence gives this technique tremendous potential in targeted therapy of a wide spectrum of both infectious and non-infectious diseases. It can find its place in the management of any condition where the removal or cleavage of specific genetic material might be beneficial. However, barely a few, if any, of the therapeutic procedures using CRISPR/Cas9 reached the state of the experimental application in human medicine. Thus, most of the discussed data shall be seen as a perspective and potential approach for future treatments.

Significant progress in this field has been made by taking advantage of cell line and animal models. Therapeutic applications for various infectious, predominantly viral diseases, monogenic disorders, or anti-cancer gene therapies have been investigated [130]. While the original role of the CRISPR/Cas system in bacteria was an antiviral defense, the attempts to utilize it as an antiviral agent are not surprising. The possibility of direct targeting of viral genomes predisposes CRISPR/Cas9 system to be a promising treatment against a wide spectrum of viruses. It has been shown in cultured cells that it can restrict the viral life cycle by introducing sequence-specific breaks in essential parts of the viral genome [131–133]. Based on the specific virus biology, different life stages can be targeted. For example, covalently closed circular DNA (cccDNA) of HBV is the preferred target while it is not reduced by standard treatment with nuclear analogs [134]; on the other hand, CRISPR/Cas9 treatment of HIV aims at integrated provirus removal or inactivation [135]. Similarly, in the case of HSV-1, replication of the virus can be effectively impaired by using nucleoside analogs; however, the latent virus remains unaffected. Targeting key viral genes by CRISPR/Cas9 leads to indels resulting in reduced infectivity or even completely abrogated infection. Resistant strains of escaped mutants were fully prevented by simultaneously targeting two essential viral genes by co-delivery of two gRNAs [136, 137]. When multiplexed CRISPR/Cas9 was applied to target the latent EBV genomes in a Burkitt’s lymphoma patient-derived B cell line, an eradication or significant reduction of viral load in the majority of infected cells resulted in a dramatic cell proliferation arrest and induction of apoptosis [138, 139]. In the case of HPV, already established HPV-driven tumors have to be targeted. The CRISPR/Cas9 system was employed to inactivate the viral oncogenes E6 and/or E7, thereby restoring p53/Rb levels and induction of apoptosis of HPV16-infected cells [140]. Direct targeting of RNA viruses (e.g., HCV) is also possible [48]. It is not surprising, that after the outbreak of COVID-19, several research groups attempted to adjust the CRISPR/Cas technology as a potential anti-SARS-CoV-2 therapeutics [141–143].

Another group of diseases that can be treated with the utilization of CRISPR/Cas are monogenic disorders caused by single gene defects. Therapies for this group of diseases are generally limited to managing symptoms regardless of the pathogenic mutation causing the disease in the patient. CRISPR/Cas system represents a promising personalized gene-editing approach in the treatment of monogenic disorders. The results of preclinical studies carried out on human stem cells, patient-derived induced pluripotent stem cells (iPSCs), or animal models showed successful correction of various types of mutations in defective genes causing monogenic diseases such as cystic fibrosis [144, 145], Duchenne muscular dystrophy [146, 147], hemophilia [148, 149], Huntington’s disease [150–152], and sickle cell anemia [153–155].

CRISPR/Cas system represents a powerful perspective tool with a wide spectrum of use in PPP medicine. The greatest possibilities of using a CRISPR-based therapeutic approach are in personalized cancer treatment. The potential of CRISPR-based screening combined with high-throughput next-generation sequencing brings great possibilities for discovering new potential therapeutic targets, biomarkers for early diagnosis of disease, monitoring of disease progress, therapy efficiency, and prognostic biomarkers [156]. Drug and immunotherapy resistance is a big problem in long-term treatment, predominantly in oncology. CRISPR/Cas system can also be utilized to identify genes and mutations responsible for drug resistance and thereby help with individualized, effective therapy [157].

CRISPR/Cas mechanism can be utilized in personalized therapy not only for gene editing and regulation but also for editing of epigenetic modification, which is one of the gene expression activation or silencing mechanisms. In tumors, oncogenes and tumor suppressor genes are often regulated via epigenetic modifications, which are also possible therapeutic targets for personalized medicine. For epigenetic editing, the most promising technology was developed based on the fusion of deactivated Cas9 protein (dCas9) without endonuclease activity serving as DNA-binding protein with an epigenetic effector (epi-effector) [158] as described above. There are several studies bringing promising results of epigenetic modulation of tumor suppressor genes or oncogenes through dCas9/effector complexes in the most common cancers, for example, activation of *PTEN* in breast cancer [159], *BRAF1* gene in cervical cancer [160] and other genes in colon cancer [161], liver cancer [162], prostate cancer [163] or lung cancer [164] and others.

CRISPR/Cas-based technology also offers opportunities for advances in immunotherapy based on artificial CAR- (chimeric antigen receptor) T cells recognizing specific tumor-associated antigens. This method brings innovative, precise engineering for reprogramming patients’ own T cells to target tumor cells. Next-generation CAR T cells engineered with CRISPR/Cas9 system help overcome CAR-T cell therapy’s limitations such as stability, low persistence, T cell depletion, or toxic tumor microenvironment and open the possibilities for personalized immunotherapy not only for hematopoietic malignancies but also for solid tumors [165]. CRISPR/Cas9 is an effective tool for the knock-out of genes responsible for producing negative regulators of T cells like PD-1 or CTLA-4, Fas signaling, and toxic cytokines, which are factors reducing the effectiveness of CAR T cell therapy. These targets were successfully disrupted with a multiplex CRISPR/Cas9 gene-editing approach to increase the success of therapy [166–168]. CRISPR technology improves also the targeting of CAR encoding cassette to the specific location under the control of the appropriate promotor to ensure stable expression and prevents unspecific insertion observed in the case of viral vectors [165, 169, 170].

## Outlook in the framework of 3P medicine

### Predictive, preventive, and personalized approach in primary care—ethical aspects

Primary care considers pre-pregnancy check-up as being crucial for the cost-effective diagnostics and preventive strategies. To this end, an advanced screening of suboptimal health status to predict and prevent individual health risks prior to planned pregnancies meets needs of young populations and of the society at large and falls into the framework of 3P medicine. Contextually, pre-pregnancy check-up has been demonstrated as being pivotal for advanced health policy [7].

CRISPR/Cas is a feasible genome-editing technology to prevent transmission of parental life-threatening chrDNA mutations to offspring. To this end, human germline gene editing (HGGE) is the tool to introduce permanent genetic modification to the embryo by both*—*eliminating and introducing DNA information. Due to severe technological challenges and ethical considerations, HGGE is the subject to a detailed analysis and careful consideration by researchers, relevant scientific groups, and governmental organizations. There is a consensus amongst the majority of them that, due to a number of unanswered scientific, ethical, and policy questions, currently, it is inappropriate to perform HGGE culminating in human pregnancy. The major disagreement is about the types of research which should be allowed. For example, both*—*the European Group on Ethics in Science and New Technologies, EGE, and NIH in the USA (which does not fund any use of gene-editing technologies in human embryos) suggested a partial or full moratorium on HGGE research, whereas the US National Academy of Sciences and National Academy of Medicine supports basic and preclinical HGGE research [171]. Due to a limited access to the human germline material and severe ethical concerns, stem cells are suggested as the reasonable alternative model for the HGGE approaches to promote the field [172].

### Technological challenges of gene editing in the mitochondrial disease

The “gatekeeper” role of mitochondria is demonstrated for the wide range of molecular, cellular, organ, and organismal functions. Compromised mitochondrial health is linked to systemic effects in multi-organ functionality. Consequently, mitochondrial health sustainability is pivotal for primary, secondary, and tertiary care [173, 174].

Mitochondrial disease (MD) is a devastating inborn pathology originating from defects either directly in mtDNA or/and in chrDNA encoding for proteins localized to mitochondria. The most severe forms of MD are most frequently linked to OXPHOS function impairments, which are fatal causing death of affected newborn infants shortly after their birth. Although many mothers are at risk of transmitting MD to their offspring, corresponding screening programs are still underdeveloped and there is no cure of MD. To this end, mitochondrial replacement therapy, and CRISPR/Cas are considered promising technological solutions to prevent MD transmission from the affected mother to offspring [175].

Being proven as a highly effective system for the nuclear genome editing, CRISPR/Cas feasibility for mitochondrial genome editing is, however, controversial [176]. Specifically, the delivery of this gene-editing system into mitochondria remains debatable. RNA import process into mitochondria is insufficiently understood and the mitochondrial DNA repair machinery differs substantially from this of the chrDNA. To this end, the basis of the CRISPR/Cas-associated editing system, namely DNA double-strand break repair, is lacking in mitochondria, in contrast to the homologous recombination-mediated repair mechanisms that are detectable there.

Consequently, CRISPR/Cas editing system application in regard to the MD is focused mainly on studying mitochondrial biology and genome-related disorders being currently restricted to distinctive tasks:

- Ex vivo MD modeling
- Creation of isogenic populations of diseased cells with homogenous phenotype, e.g., with clearly defined mutations causing co-enzyme Q10 deficiency to distinguish individual phenotypes in MD
- A genome-wide library screening to investigate the relevance of individual genes to the co-enzyme Q10 biosynthesis machinery, ATP-production versus deficiency as well as to the electron-chain transfer efficacy.

These models utilizing CRISPR/Cas editing system are considered highly efficient to investigate potential therapeutic treatments tailored to the individualized profile of the MD-affected patients discriminating between moderate and severe medical conditions [176].

## Data Availability

Not applicable.

## Abbreviations

AIOD-CRISPR: All-In-One dual CRISPR/Cas12a
CASFISH: Cas9-mediated fluorescence in situ hybridization
CEP290: Centrosomal protein 290
CRISPR/Cas: Clustered Regularly Interspaced Short Palindromic Repeats/CRISPR-associated sequences
CRISPRi: CRISPR interference
crRNA: CRISPR RNA
dCas9: Dead or deactivated Cas9
DETECTR: DNA endonuclease- targeted CRISPR trans reporter
enChIP: Engineered DNA-binding molecule-mediated chromatin immunoprecipitation
EPMA: European Association for Predictive, Preventive and Personalised Medicine
FnCas9: Cas9 molecules of *Francisella novicida*
FokI: Restriction endonuclease found in *Flavobacterium okeanokoites*
gRNA: Guide RNA
HDR: Homology-directed repair
HGGE: Human germline gene editing
KRAB: Krüppel associated box
lncRNAs: Long non-coding RNAs
NASBA: Nucleic acid sequence-based amplification
NHEJ: Non-homologous end joining pathway
PAM: Protospacer adjacent motif
PPPM or 3PM: Predictive preventive personalized medicine
p300: E1A-binding protein p300
RPA: Recombinase polymerase amplification
scFv: Single-chain variable fragment
sgRNA: Single chimeric guide RNA
SHERLOCK: Specific High-sensitivity Enzymatic Reporter un-Locking
tracrRNA: Trans-activating CRISPR RNA
